# Multifaceted anti-amyloidogenic and pro-amyloidogenic effects of C-reactive protein and serum amyloid P component *in vitro*

**DOI:** 10.1038/srep29077

**Published:** 2016-07-06

**Authors:** Daisaku Ozawa, Ryo Nomura, P. Patrizia Mangione, Kazuhiro Hasegawa, Tadakazu Okoshi, Riccardo Porcari, Vittorio Bellotti, Hironobu Naiki

**Affiliations:** 1 Life Science Unit, Tenure-Track Program for Innovative Research, University of Fukui, Fukui 910-1193, Japan; 2 Department of Molecular Pathology, Faculty of Medical Sciences, University of Fukui, Fukui 910-1193, Japan; 3 Wolfson Drug Discovery Unit, Centre for Amyloidosis and Acute Phase Proteins, Division of Medicine, University College London, London NW3 2PF, UK

## Abstract

C-reactive protein (CRP) and serum amyloid P component (SAP), two major classical pentraxins in humans, are soluble pattern recognition molecules that regulate the innate immune system, but their chaperone activities remain poorly understood. Here, we examined their effects on the amyloid fibril formation from Alzheimer’s amyloid β (Aβ) (1-40) and on that from D76N β_2_-microglobulin (β2-m) which is related to hereditary systemic amyloidosis. CRP and SAP dose-dependently and substoichiometrically inhibited both Aβ(1-40) and D76N β2-m fibril formation in a Ca^2+^-independent manner. CRP and SAP interacted with fresh and aggregated Aβ(1-40) and D76N β2-m on the fibril-forming pathway. Interestingly, in the presence of Ca^2+^, SAP first inhibited, then significantly accelerated D76N β2-m fibril formation. Electron microscopically, the surface of the D76N β2-m fibril was coated with pentameric SAP. These data suggest that SAP first exhibits anti-amyloidogenic activity possibly via A face, followed by pro-amyloidogenic activity via B face, proposing a model that the pro- and anti-amyloidogenic activities of SAP are not mutually exclusive, but reflect two sides of the same coin, i.e., the B and A faces, respectively. Finally, SAP inhibits the heat-induced amorphous aggregation of human glutathione S-transferase. A possible role of pentraxins to maintain extracellular proteostasis is discussed.

C-reactive protein (CRP) and serum amyloid P component (SAP) are the two major classical pentraxins in humans^1^,^2^,^3^ (Fig. 1a,b). They have a unique pentameric structure and bind to their ligands calcium-dependently with their B faces. CRP and SAP are soluble pattern recognition molecules that recognize various molecules of pathogenic bacteria and damaged cells first^1^. They next interact with the complement pathway and Fcγ receptors to activate the innate immune system^1^. Human CRP is a potent acute phase protein and during inflammation, the plasma CRP concentration may increase from less than 50 μg/L to more than 500 mg/L (4.3 μM in pentamer)^3^. By contrast, human SAP is constitutively produced in the liver at an average serum concentration of 43 mg/L (0.34 μM in pentamer) in normal men and 33 mg/L in normal women^4^.

CRP is localized at various types of inflammatory sites both in humans and experimental animals^5^,^6^,^7^. Agrawal’s group reported that at acidic pH *in vitro*, pentameric CRP bound calcium-independently to various types of proteins with altered conformations including amyloid β (Aβ)^8^,^9^. Based on the fact that the pH at the inflammatory sites generally becomes acidic^10^,^11^,^12^, they hypothesized that pentameric CRP protects against toxic conditions caused by protein misfolding and aggregation in acidic inflammatory environments, but they showed no data on the anti-amyloidogenic, anti-aggregation activity of CRP. Consistent with their hypothesis, CRP immunoreactivity was recognized in the senile plaques and neurofibrillary tangles in the brain of Alzheimer’s disease patients^13^,^14^,^15^.

SAP is present universally in all extracellular amyloid deposits^2^. Its primary role in amyloidogenesis is thought to enhance the formation and deposition of amyloid fibrils by binding to the surface of amyloid fibrils calcium-dependently with the B face, protecting them from proteolytic degradation and stabilizing their structure^16^,^17^,^18^,^19^,^20^. SAP exists as a single uncomplexed pentamer in whole serum and enough calcium is available for the binding of SAP to amyloid fibrils^21^. By contrast, Janciauskiene *et al.*^22^ reported that SAP inhibits Aβ amyloid fibril formation in a calcium-free condition *in vitro*. Moreover, using a cell culture system and a Drosophila model for transthyretin (TTR)-associated amyloidosis, Andersson *et al.*^23^ revealed the protective effect of SAP on TTR-induced cell death and degenerative phenotypes. The new role they proposed was that SAP attenuates the toxicity of early amyloidogenic aggregates. However, both groups did not determine the face or domain of SAP responsible for the anti-amyloidogenic, anti-toxic activities. Coker *et al.*^24^ reported that human SAP calcium-independently enhances the refolding yield of denatured lactate dehydrogenase and protects against enzyme inactivation during agitation of dilute solutions. They suggested that the A face and/or the edge of the SAP pentamer has chaperone activity not the amyloid recognition site on the B face of SAP. However, they showed no data on the anti-amyloidogenic, anti-aggregation activity of the A face of SAP. No convincing data or models have been published thus far to explain the discrepancy between the pro- and anti-amyloidogenic activities of SAP.

Wilson’s group proposed that a small but expanding family of constitutively secreted extracellular chaperones (ECs) may act as both sensors and clearance mediators of misfolded proteins in extracellular fluids, thereby inhibiting the manifestation of various amyloidoses caused by the misfolding and aggregation of extracellular proteins^25^,^26^,^27^. We recently reported that α_2_-macroglobulin (α2M), a representative extracellular chaperone, substoichiometrically inhibits β_2_-microglobulin (β2-m) amyloid fibril formation *in vitro* and suggested that under conditions where native β2-m is partially unfolded, tetrameric α2M is also converted to dimeric form with exposed hydrophobic surfaces to favor the hydrophobic interaction with unfolded β2-m^28^.

In this paper, we report that the pentraxins CRP and SAP inhibit the amyloid fibril formation from Alzheimer’s Aβ(1-40) and D76N β2-m, the latter of which causes hereditary systemic amyloidosis^29^. Importantly, SAP exhibited the anti-amyloidogenic activity first, followed by a pro-amyloidogenic phase on D76N β2-m amyloid fibril formation in the presence of Ca^2+^, which could clarify the apparent discrepancy among various reports. A possible role of pentraxins to maintain extracellular proteostasis will be discussed.

## Results

### Evaluation of the assembly states of CRP and SAP with and without the physiological concentration of Ca^2+^

**Figure 1:**
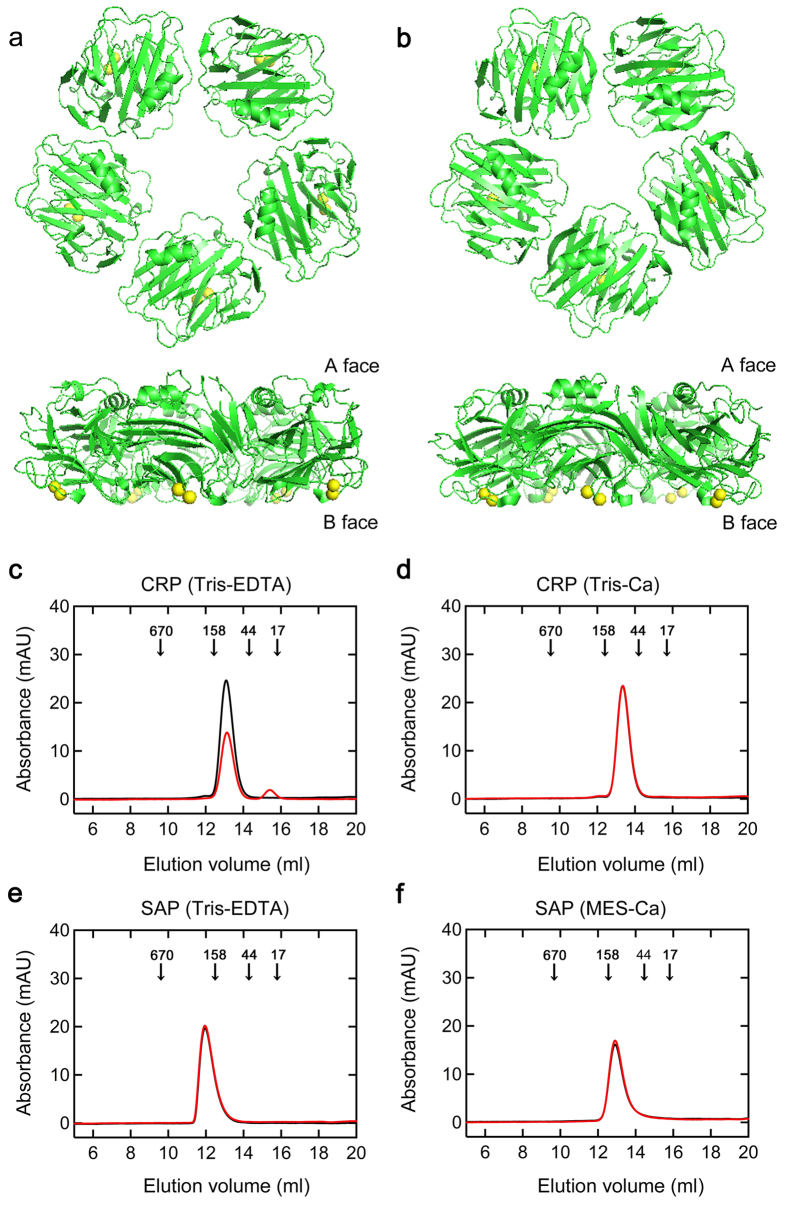
Structure and assembly states of CRP and SAP. (**a,b**) Structures of pentameric CRP and SAP. These figures were prepared with the PDB files 1B09 for CRP (**a**) and 1SAC for SAP (**b**) using the software PyMOL. Pentamers were viewed along the 5-fold axis of symmetry from the A face (upper) and perpendicular to the 5-fold axis (lower). Yellow: calcium ions. (**c–f**) Analysis of molecular weight distribution of CRP and SAP by gel filtration chromatography. CRP (**c,d**) and SAP (**e,f**) at 1.5 μM were incubated in Tris-EDTA (pH 7.5) (**c,e**), Tris-Ca (pH 7.5) (**d**), or MES-Ca buffer (pH 7.0) (**f**) at 37 °C for 0 (black line) or 72 h (red line), then 300 μL aliquots were applied on a column equilibrated and eluted with the same buffer at 15 °C. Elution was monitored by absorbance at 280 nm. Arrows in each figure indicate the elution volumes of molecular weight markers (kDa).

We first evaluated the assembly states of CRP and SAP in various buffer conditions (see Methods). In the presence of Ca^2+^ (Fig. 1d), the pentameric structure of CRP was stable even after 72-h incubation at 37 °C. On the other hand, when CRP was incubated at 37 °C for 72 h in Ca^2+^-free Tris-EDTA buffer (Fig. 1c), the reaction mixture became turbid and a small monomer peak (23 kDa) was observed. This indicates that the pentameric structure of CRP may become unstable at 37 °C in Ca^2+^-free buffer, causing disassembly of some pentamers into monomers and their nonspecific aggregation. SAP assembled into the stable decamers (255 kDa) in Ca^2+^-free Tris-EDTA buffer (Fig. 1e). Recently, Coker *et al.*^30^ reported that the X-ray crystal structure of calcium-free SAP shows a B-face-to-B-face decamer with the protease sensitive loops from the calcium-binding site extended to clasp the opposite pentamer. Thus, in the following experiments, we used this non-physiological EDTA-decamer to check the anti-amyloidogenic activity of the A face of SAP. Isolated human SAP undergoes rapid autoaggregation when exposed to Ca^2+^ 31. Thus, based on the report that 500 to 750 mM NaCl keeps SAP pentameric even in the presence of Ca^2+^ 32, we prepared MES-Ca buffer, comprised of 50 mM MES-NaOH (pH 7.0), 500 mM NaCl, and 2 mM CaCl_2_. In this buffer condition (Fig. 1f), SAP remained pentameric (127 kDa) even after 72-h incubation at 37 °C.

### CRP and SAP inhibit the Aβ(1-40) amyloid fibril formation in a Ca^2+^-independent manner

**Figure 2:**
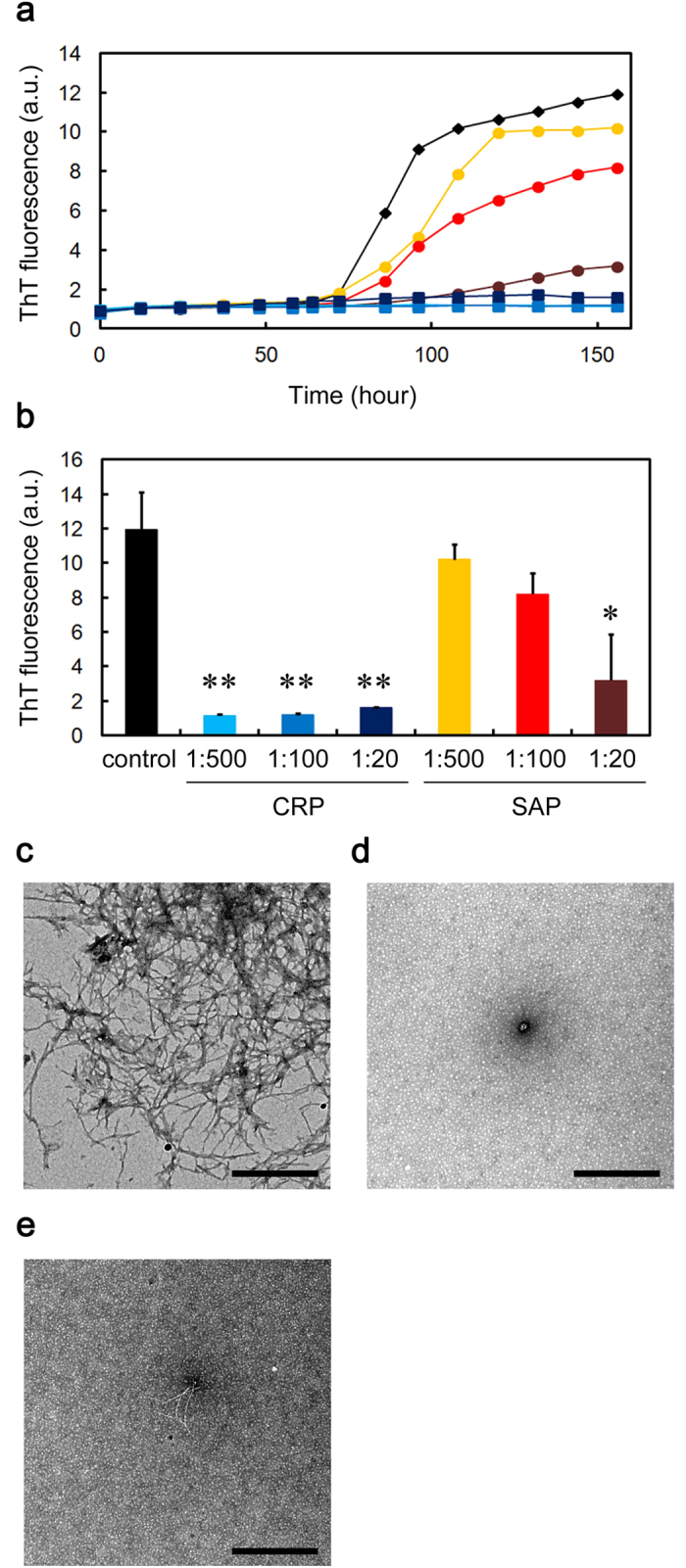
Effects of CRP and SAP on Aβ(1-40) amyloid fibril formation in Ca^2+^-free Tris-EDTA buffer. (**a**) Time course of fibril formation monitored by ThT fluorescence in the absence (black diamond) or presence of 1:500 (molar ratio of CRP to Aβ(1-40)) (light blue square), 1:100 (blue square), or 1:20 (dark blue square) CRP, or 1:500 (orange circle), 1:100 (red circle), or 1:20 (dark brown circle) SAP. Each point represents the average of three independent incubations. Representative data of three independent experiments are shown. (**b**) ThT fluorescence of each sample at 156 h in (**a**). The data are mean ± SD of three independent incubations. Statistical analysis was performed by unpaired Student’s t-test. *P < 0.05, **P < 0.01 vs. control. (**c–e**) Electron microscopy images of the samples of fibril formation. The sample prepared in the absence (**c**) or presence of 1:20 CRP (d) or SAP (**e**) was incubated at 37 °C for 120 h. The scale bar represents 0.5 μm.

**Figure 3:**
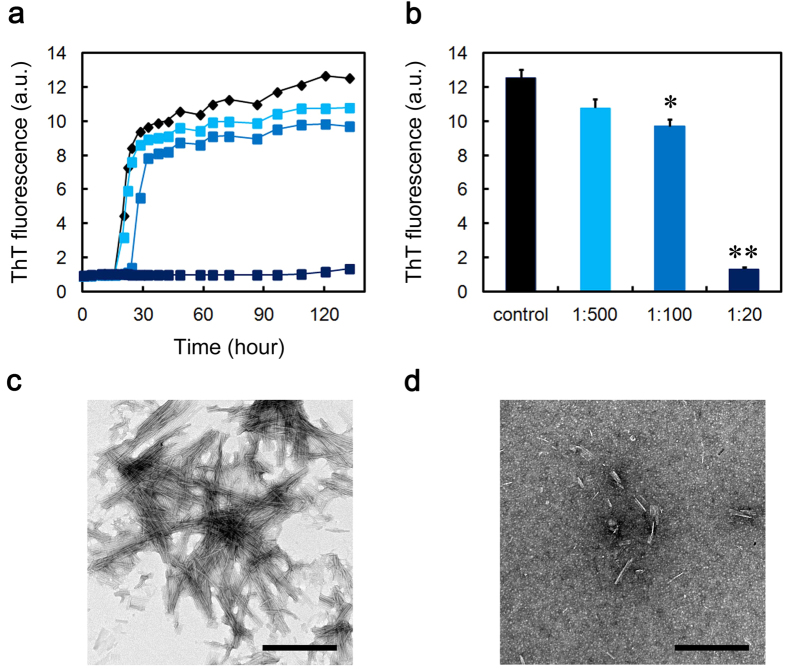
Effect of CRP on Aβ(1-40) amyloid fibril formation in Tris-Ca buffer. (**a**) Time course of fibril formation monitored by ThT fluorescence in the absence (black diamond) or presence of 1:500 (molar ratio of CRP to Aβ(1-40)) (light blue square), 1:100 (blue square), or 1:20 (dark blue square) CRP. Each point represents the average of three independent incubations. Representative data of three independent experiments are shown. (**b**) ThT fluorescence of each sample at 132 h in (**a**). The data are mean ± SD of three independent incubations. Statistical analysis was performed by unpaired Student’s t-test. *P < 0.05, **P < 0.01 vs. control. (**c,d**) Electron microscopy images of the samples of fibril formation. The sample prepared in the absence (**c**) or presence of 1:20 CRP (**d**) was incubated at 37 °C for 120 h. The scale bar represents 0.5 μm.

**Figure 4:**
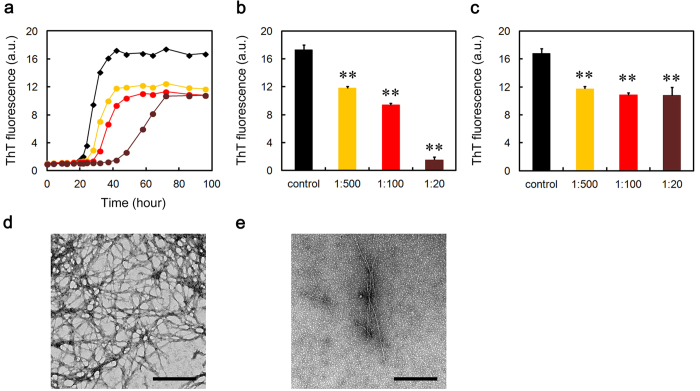
Effect of SAP on Aβ(1-40) amyloid fibril formation in MES-Ca buffer. (**a**) Time course of fibril formation monitored by ThT fluorescence in the absence (black diamond) or presence of 1:500 (molar ratio of SAP to Aβ(1-40)) (orange circle), 1:100 (red circle), or 1:20 (dark brown circle) SAP. Each point represents the average of three independent incubations. Representative data of three independent experiments are shown. (**b,c**) ThT fluorescence of each sample at 42 (**b**) or 96 h (**c**) in (**a**). The data are mean ± SD of three independent incubations. Statistical analysis was performed by unpaired Student’s t-test. **P < 0.01 vs. control. (**d,e**) Electron microscopy images of the samples of fibril formation. The sample prepared in the absence (**d**) or presence of 1:20 SAP (**e**) was incubated at 37 °C for 48 h. The scale bar represents 0.5 μm.

We next examined the effects of CRP and SAP on Aβ(1-40) amyloid fibril formation in three different buffer conditions. Thioflavin T (ThT) assay and electron microscopy revealed that SAP dose-dependently and substoichiometrically inhibited amyloid fibril formation of Aβ(1-40) incubated in Ca^2+^-free Tris-EDTA buffer (pH 7.5) (Fig. 2). CRP almost completely inhibited fibril formation even at 1:500 molar ratio of CRP to Aβ(1-40) (Fig. 2). As calcium-free SAP exhibits a B-face-to-B-face decamer (Fig. 1e)^30^, these data may indicate that the A-face of SAP may have anti-amyloidogenic activity in a Ca^2+^-independent manner. Similarly, ThT assay and electron microscopy revealed that in the presence of the physiological concentration of Ca^2+^, both pentameric CRP and pentameric SAP dose-dependently and substoichiometrically inhibited amyloid fibril formation (Figs 3 and 4). At the final equilibrium point of Fig. 4a (96 h), SAP had no dose-dependent inhibitory effect and ThT fluorescence elevated to 64 to 70% of the control in all SAP concentrations examined (Fig. 4c).

In the presence of Ca^2+^, the lag time of ThT fluorescence kinetics decreased to about one third of that in the absence of Ca^2+^ (about 20 h in Figs 3a and 4a vs. about 60 h in Fig. 2a), suggesting that Ca^2+^ enhanced the fibrillar aggregation of Aβ(1-40). Other groups reported that physiological concentrations of Ca^2+^ accelerate Aβ amyloid fibril formation by increasing the aggregation of early forming protofibrillar structures and markedly increasing conversion of protofibrils to mature amyloid fibrils^33^, as well as by enhancing both the elongation rate and the stability of amyloid fibrils^34^. In the presence of Ca^2+^, the constituent of buffers (i.e., Tris-Ca vs. MES-Ca) affected the fibril morphology (compare Figs 3c and 4d). Interestingly, in the presence of Ca^2+^, CRP and SAP were less effective than in the absence of Ca^2+^ in preventing the aggregation of Aβ(1-40). In the absence of Ca^2+^, CRP at 1:500 almost completely inhibited the increase in ThT fluorescence but exhibited no significant inhibitory activity in the presence of Ca^2+^ (compare Figs 2b and 3b). Similarly, in the absence of Ca^2+^, SAP at 1:20 lowered ThT fluorescence to 26.7 ± 22.4% of the control while only to 64.2 ± 6.8% in the presence of Ca^2+^ (compare Figs 2b and 4c).

### CRP and SAP inhibit the D76N β2-m amyloid fibril formation in a Ca^2+^-independent manner

We next examined the effects of CRP and SAP on D76N β2-m amyloid fibril formation in three different buffer conditions. When D76N β2-m was incubated in Ca^2+^-free Tris-EDTA buffer (pH 7.5), both CRP and SAP dose-dependently and substoichiometrically inhibited amyloid fibril formation as evaluated by ThT assay and electron microscopy (Supplementary Fig. S1). Similarly, in the presence of the physiological concentration of Ca^2+^, pentameric CRP dose-dependently and substoichiometrically inhibited amyloid fibril formation (Supplementary Fig. S2).

### SAP exhibits the anti-amyloidogenic activity first, followed by a pro-amyloidogenic phase on D76N β2-m amyloid fibril formation in MES-Ca buffer

**Figure 5:**
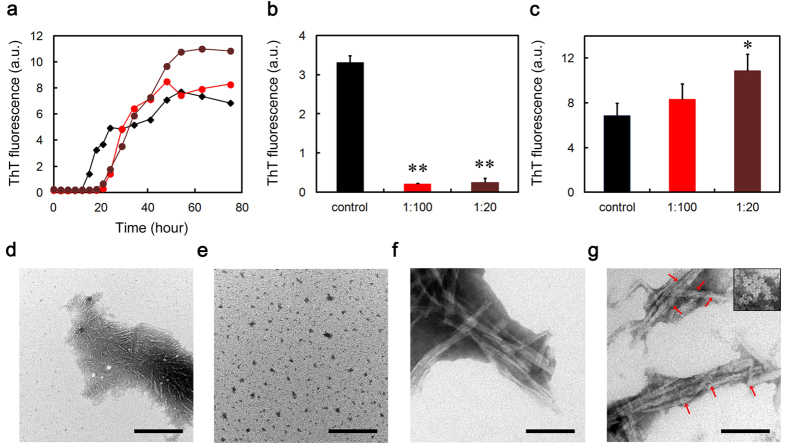
Effect of SAP on D76N β2-m amyloid fibril formation in MES-Ca buffer. (**a**) Time course of fibril formation monitored by ThT fluorescence in the absence (black diamond) or presence of 1:100 (molar ratio of SAP to D76N β2-m) (red circle), or 1:20 (dark brown circle) SAP. Each point represents the average of three independent incubations. Representative data of three independent experiments are shown. (**b,c**) ThT fluorescence of each sample at 18 (**b**) or 75 h (**c**) in (**a**). The data are mean ± SD of three independent incubations. Statistical analysis was performed by unpaired Student’s t-test. *P < 0.05, **P < 0.01 vs. control. (**d–g**) Electron microscopy images of the samples of fibril formation. The sample prepared in the absence (**d,f**) or presence of 1:20 SAP (**e,g**) was incubated at 37 °C for 18 h (**d,e**) or 75 h (**f,g**). The scale bar represents 0.5 μm (**d,e**) or 100 nm (**f,g**). In (**g**), inset indicates pentameric SAP at the same magnification and red arrows indicate pentameric SAP bound to the surface of amyloid fibrils.

As shown in Fig. 5a,b, pentameric SAP extended the lag phase of D76N β2-m amyloid fibril formation in the presence of Ca^2+^at a physiological concentration. This suggests that pentameric SAP (possibly A face) inhibited the nucleation of D76N β2-m by interacting with soluble oligomers and to some extent, with partially unfolded monomers. However, after such a prolonged lag phase, ThT fluorescence of SAP-containing samples increased more rapidly than in the absence of SAP (Fig. 5a) and at SAP:D76N β2-m = 1:20, significantly higher equilibrium fluorescence was observed as compared to the control (Fig. 5c). These observations were confirmed electron microscopically (Fig. 5d–g). Interestingly, in the SAP-containing sample at 75 h (Fig. 5g), the surfaces of amyloid fibrils were coated with doughnut-shaped pentameric SAP (13.4 nm in diameter). These data suggest that after mature amyloid fibrils were formed in the reaction mixture, high-affinity binding of the B face to the surface of amyloid fibrils predominated, leading to the enhanced amyloid fibril formation.

**Figure 6:**
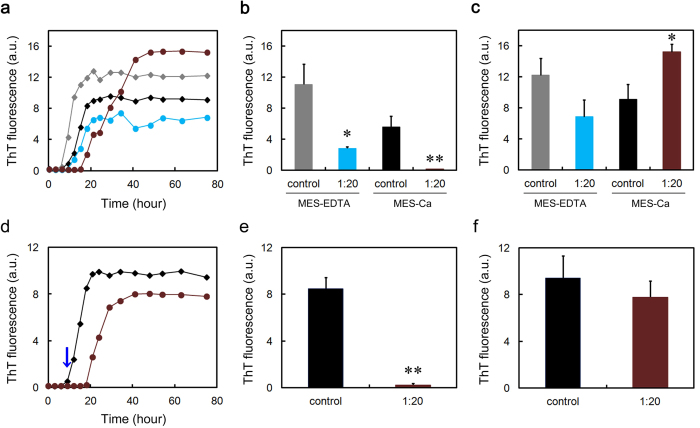
Switching-off of the pro-amyloidogenic effect of SAP. (**a**) Effect of SAP on D76N β2-m amyloid fibril formation in MES-EDTA buffer. Time course of fibril formation was monitored by ThT fluorescence in the absence (gray and black diamonds) or presence (blue and dark brown circles) of 1:20 (molar ratio of SAP to D76N β2-m) SAP in MES-EDTA (gray diamond and blue circle) or MES-Ca buffer (black diamond and dark brown circle). Each point represents the average of three independent incubations. Representative data of three independent experiments are shown. (**b,c**) ThT fluorescence of each sample at 15 (**b**) or 75 h (**c**) in (**a**). The data are mean ± SD of three independent incubations. Statistical analysis was performed by unpaired Student’s t-test. *P < 0.05, **P < 0.01 vs. control. (**d**) Effect of EDTA spiking on D76N β2-m amyloid fibril formation in the absence (black diamond) or presence (dark brown circle) of 1:20 SAP in MES-Ca buffer. EDTA at the final concentration of 10 mM was spiked to the samples at 9 h (blue arrow). Each point represents the average of three independent incubations. Representative data of three independent experiments are shown. (**e,f**) ThT fluorescence of each sample at 18 (**e**) or 75 h (**f**) in (**d**). The data are mean ± SD of three independent incubations. Statistical analysis was performed by unpaired Student’s t-test. **P < 0.01 vs. control.

To confirm the validity of this working model, we performed additional experiments. When D76N β2-m was incubated with SAP in MES-EDTA buffer, the lag phase of D76N β2-m amyloid fibril formation was significantly extended and although not significant, the final ThT fluorescence decreased to 56.3 ± 17.9% of the control (Fig. 6a–c). Moreover, when EDTA was spiked to the SAP-containing MES-Ca samples during the lag phase, the lag phase was significantly extended and although not significant, the final ThT fluorescence decreased to 82.7 ± 14.4% of the control (Fig. 6d–f). These data indicate that EDTA may switch off the pro-amyloidogenic effect of SAP by stacking it to the B-face-to-B-face decamer (Supplementary Fig. S3) but keep its anti-amyloidogenic effect intact.

We confirmed that the anti-amyloidogenic activities of CRP and SAP are significant because human α_1_-acid glycoprotein (AGP), a 41 to 43-kDa human acute phase protein^35^ which has recently been suggested as a novel member of ECs^36^, did not inhibit Aβ(1-40) and D76N β2-m amyloid fibril formation under any conditions examined (Supplementary Fig. S4).

### CRP and SAP interact with Aβ(1-40) and D76N β2-m on the fibril-forming pathway

**Figure 7:**
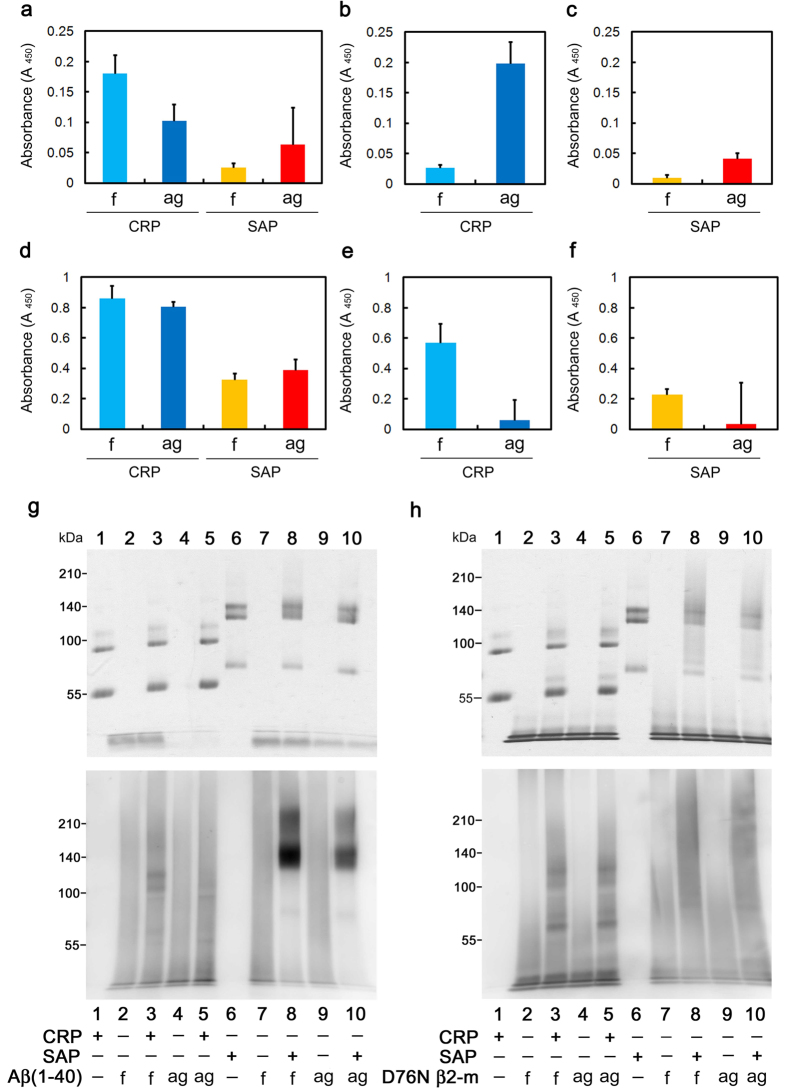
Interactions of CRP and SAP with fresh and aggregated Aβ(1-40) and D76N β2-m. (**a–f**) Binding of Aβ(1-40) (**a–c**) and D76N β2-m (**d–f**) to immobilized CRP and SAP. CRP and SAP were immobilized on an ELISA plate and incubated with fresh (**f**) and aggregated (ag) Aβ(1-40) and D76N β2-m in Tris-EDTA (**a,d**), Tris-Ca (**b,e**), or MES-Ca buffer (**c,f**). The data are mean ± SD of three independent incubations. Representative data of three independent experiments are shown. (**g,h**) Crosslinking experiments. At 0 h (fresh mixture, f) and at the beginning of the growth phase (aggregated mixture, ag), the reaction mixture containing Aβ(1-40) or D76N β2-m was spiked with 1:20 CRP and SAP, and incubated for 30 min at 37 °C. After BS^3^ was added to the mixture, SDS-PAGE (top) and western blotting analysis (bottom) were performed. In western blotting analysis, bound Aβ(1-40) and D76N β2-m were detected with anti-human Aβ(1-40) and β2-m antibodies, respectively.

A range of ECs reportedly inhibits amyloid fibril formation *in vitro* by interacting with soluble oligomeric species formed early in the aggregation pathway, rather than binding to monomeric precursor proteins or mature amyloid fibrils^25^,^26^,^27^. Yerbury *et al.*^37^,^38^ reported that prefibrillar species on the fibril-forming pathway are enriched in the reaction mixture at the beginning of the growth phase (We refer it as the aggregated mixture). Thus, we next performed an enzyme-linked immunosorbent assay (ELISA) to assess the interaction of CRP and SAP with fresh and aggregated Aβ(1-40) and D76N β2-m. As shown in Fig. 7a–f, CRP and SAP immobilized on an ELISA plate captured both fresh and aggregated Aβ(1-40) and D76N β2-m under all buffer conditions examined. As shown in Fig. 7b,c, CRP and SAP preferentially captured aggregated Aβ(1-40) in the presence of Ca^2+^, while in other cases, preferential binding of CRP and SAP to aggregated samples was not clear or convincing.

To further characterize the interaction of pentraxins with Aβ(1-40) and D76N β2-m, we performed crosslinking experiments. Crosslinking reaction was performed in the reaction mixture of Aβ(1-40) and D76N β2-m amyloid fibril formation in the presence of Ca^2+^. At 0 h (fresh mixture) and at the beginning of the growth phase (aggregated mixture), the reaction mixture containing Aβ(1-40) or D76N β2-m was spiked with 1:20 CRP and SAP. After BS^3^ was added to the mixture, SDS-PAGE and western blotting analysis were performed. Crosslinked CRP was electrophoresed as dimers and tetramers, while crosslinked SAP was electrophoresed as trimers and pentamers (Fig. 7g,h, top). As shown in Fig. 7g, bottom, Aβ(1-40) bound mainly to tetrameric CRP and pentameric SAP. Interestingly, both CRP and SAP captured fresh Aβ(1-40) more than aggregated Aβ(1-40) (compare lines 3 and 5, and lines 8 and 10, respectively). As shown in Fig. 7h, bottom, D76N β2-m bound similarly to dimeric and tetrameric CRP, as well as to trimeric and pentameric SAP. Both CRP and SAP captured fresh and aggregated D76N β2-m similarly (compare lines 3 and 5, and lines 8 and 10, respectively). Although our experimental system cannot discriminate monomers from oligomers captured by pentraxins, these data suggest that pentraxins could capture both monomeric and oligomeric Aβ(1-40) and D76N β2-m.

### SAP inhibits the heat-induced amorphous aggregation of glutathione S-transferase (GST)

**Figure 8:**
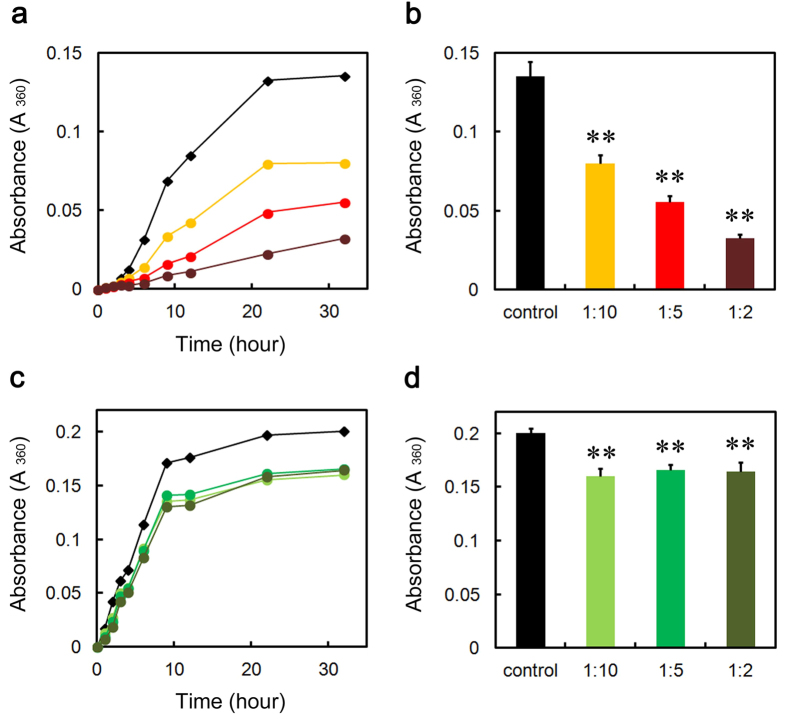
Effect of SAP on heat-induced GST aggregation in MES-Ca and Tris-EDTA buffers. (**a,c**) Time course of GST aggregation monitored by turbidity in the absence (black diamond) or presence of 1:10 (molar ratio of SAP to GST) (orange circle), 1:5 (red circle), or 1:2 (dark brown circle) SAP in MES-Ca buffer (**a**), or 1:10 (right green circle), 1:5 (green circle), or 1:2 (dark green circle) SAP in Tris-EDTA buffer (**c**) at 43 °C. Each point represents the average of three independent incubations. Representative data of three independent experiments are shown. (**b,d**) Turbidity of each sample at 32 h in MES-Ca (**b**) and Tris-EDTA buffers (**d**). The data are mean ± SD of three independent incubations. Statistical analysis was performed by unpaired Student’s t-test. **P < 0.01 vs. control.

Amyloid fibrils and amorphous aggregates are two types of aberrant aggregates associated with protein misfolding diseases^39^. Thus, we finally assessed the effect of SAP on heat-induced amorphous aggregation of GST (47 kDa), malate dehydrogenase (MDH) (70 kDa), and lactate dehydrogenase (LDH) (140 kDa) in MES-Ca and Tris-EDTA buffers. We did not examine the effect of CRP because CRP autoaggregated at 43 °C. When GST was incubated in MES-Ca buffer (pH 7.0), pentameric SAP dose-dependently and substoichiometrically inhibited GST aggregation as evaluated by turbidity assay (Fig. 8a,b). Although decameric SAP significantly and substoichiometrically inhibited GST aggregation in Ca^2+^-free Tris-EDTA buffer (pH 7.5), its effect was marginal and not dose-dependent (Fig. 8c,d). We have no clear explanation for the difference of SAP effects on GST aggregation between MES-Ca and Tris-EDTA buffers. SAP did not inhibit the aggregation of MDH and LDH even at the molar ratio of 2:1 to MDH/LDH (Supplementary Fig. S5).

## Discussion

The present study showed that CRP and SAP dose-dependently and substoichiometrically inhibit both Aβ(1-40) and D76N β2-m amyloid fibril formation in a Ca^2+^-independent manner. CRP and SAP interacted with fresh and aggregated Aβ(1-40) and D76N β2-m on the fibril-forming pathway. Interestingly, in the presence of Ca^2+^, SAP first inhibited, then significantly accelerated D76N β2-m amyloid fibril formation.

In Ca^2+^-free Tris-EDTA buffer, both pentameric CRP and decameric SAP potently inhibited Aβ(1-40) and D76N β2-m amyloid fibril formation (Figs 1 and 2 and Supplementary Fig. S1). CRP and SAP share 51% amino acid identity and very similar pentameric structures^1^. They absolutely require Ca^2+^ to bind to various molecules of pathogenic bacteria and damaged cells with their B faces^1^. Considering the B-face-to-B-face topology of SAP decamer in Tris-EDTA buffer^30^, the A faces (and/or the edges) of CRP and SAP may have the anti-amyloidogenic activity *in vitro*.

Our ELISA and crosslinking experiments suggested that pentraxins could capture both monomeric and oligomeric Aβ(1-40) and D76N β2-m (Fig. 7). A range of ECs, such as clusterin^37^,^40^,^41^, α2M^38^,^42^, and haptoglobin^38^, reportedly inhibits amyloid fibril formation *in vitro* by interacting with soluble oligomeric species formed early in the aggregation pathway, rather than binding to monomeric precursor proteins or mature amyloid fibrils. The structural basis for the interaction of ECs with their client proteins is not fully elucidated, but it is generally believed that the hydrophobic surface domains of ECs may interact with the exposed hydrophobic residues of oligomers^27^. CRP and SAP immobilized on an ELISA plate preferentially captured aggregated Aβ(1-40) in the presence of Ca^2+^ (Fig. 7b,c), while in the crosslinking experiment, both CRP and SAP captured fresh Aβ(1-40) more than aggregated Aβ(1-40) in the presence of Ca^2+^ (Fig. 7g). Although we have no clear explanation for this discrepancy, one possible reason could be because the principles of ELISA and crosslinking experiment are different from each other. However, it is worth noting that we did detect the interaction of pentraxins with fresh Aβ(1-40) and D76N β2-m in all conditions of both ELISA and crosslinking experiments, suggesting the unique interaction mode of pentraxins with client proteins.

Consistent with our data, activated α2M was reported to interact with monomeric amyloidogenic proteins^28^,^42^,^43^. Mettenburg *et al.*^43^ reported that monomeric Aβ(1-40) binds selectively to α2M that has been induced to undergo conformational change by reaction with methylamine. They also proposed that a single sequence, with amino acids 1314–1365 at the center, may be a cryptic Aβ-binding site in the native α2M, which is exposed to the solvent only after α2M is activated. We recently reported that α2M substoichiometrically inhibits the β2-m fibril formation at a neutral pH in the presence of 0.5 mM SDS^28^. Interestingly, SDS dissociated tetrameric α2M into dimers with increased surface hydrophobicity. We proposed that under conditions where native β2-m is partially unfolded and prone to aggregate (e.g., in the presence of SDS or at an acidic pH), tetrameric α2M is also converted to dimeric form with exposed hydrophobic surfaces to favor the hydrophobic interaction with unfolded β2-m, thus potently inhibiting β2-m amyloid fibril formation. Very recently, Wyatt *et al.*^42^ confirmed the validity of this model under more physiologically relevant conditions. They showed that hypochlorite, an oxidant generated *in vivo* by the innate immune system, markedly increases the anti-amyloidogenic activity of α2M by generating species, particularly dimers formed by dissociation of the native tetramer, which have enhanced surface hydrophobicity. They also demonstrated that hypochlorite-modified α2M forms the stable soluble complexes with unfolded or misfolded client proteins, including native monomeric Aβ(1-42) as well as oxidized oligomeric Aβ(1-42), thus inhibiting Aβ amyloid fibril formation. All these data indicate that in addition to the oligomeric species, monomeric amyloidogenic proteins may also be the client species for ECs.

CRP and SAP exhibited unique client specificities. First, in the presence of Ca^2+^, where Aβ(1-40) aggregated faster than in the absence of Ca^2+^, pentraxins were less effective than in the absence of Ca^2+^ in preventing the aggregation of Aβ(1-40) (see Figs 2, 3, 4). Second, in terms of molar stoichiometry, SAP was less effective in preventing the heat-induced fast aggregation of GST, compared to its ability to prevent slow fibrillar aggregation of Aβ(1-40) and D76N β2-m (compare Figs 2,4 and 5, Supplementary Fig. S1 and Fig. 8). Finally, SAP inhibited the heat-induced amorphous aggregation of GST (47 kDa) but did not inhibit the aggregation of larger client proteins, MDH (70 kDa) and LDH (140 kDa) (Fig. 8 and Supplementary Fig. S5). Carver *et al.*^44^ indicated that different ECs exhibit different efficiencies when interacting with target proteins aggregating at varying rates and ECs generally prefer to interact with slowly aggregating target proteins. They proposed that some ECs may be specific for aggregating proteins on their amorphous aggregation pathway whereas other ECs may have a preference for more slowly aggregating proteins on the amyloid fibril-forming pathway^44^. On the other hand, Niwa *et al.*^45^ evaluated the effects of the major Escherichia coli chaperones, DnaK/DnaJ/GrpE and GroEL/GroES, on ~800 aggregation-prone cytosolic E. coli proteins, using a reconstituted chaperone-free translation system. Interestingly, they found that DnaK/DnaJ/GrpE is effective for larger proteins (>60 kDa) and GroEL/GroES is biased toward 20 ~ 50 kDa proteins, indicating that the molecular weights of the client proteins may be one of the key properties for the chaperone preferences. Thus, it may be reasonable to consider that pentraxins may be more effective against smaller and slower aggregating client proteins, e.g., Aβ(1-40) (4.3 kDa) and D76N β2-m (12 kDa).

In this paper, we did not address the question whether the domain of the ATP-independent refolding activity within the A face of SAP^24^ is identical to the domain of the anti-amyloidogenic activity. Stull *et al.*^46^ characterized the kinetic mechanism of the folding of immunity protein 7 (Im7; 10 kDa) by the small ATP-independent chaperone Spy (31 kDa) from Escherichia coli. They found that Spy rapidly associates with monomeric Im7, eliminates its potential for aggregation, and allows it to fully fold into the native state while it remains bound to the surface of Spy. Future studies are essential to definitively localize both anti-amyloidogenic and refolding activities of SAP to the specific domain(s) of the A face.

The most significant contribution of this study to the amyloid science is the resolution of the controversy over the pro- and anti-amyloidogenic activities of SAP. Although many research groups reported that SAP enhances the formation and deposition of amyloid fibrils by binding to the surface of amyloid fibrils calcium-dependently with the B face^2^,^16^,^17^,^18^,^19^,^20^, other groups reported anti-amyloidogenic and anti-cytotoxic activities of SAP^22^,^23^. Consistent with the observation that SAP inhibits Aβ amyloid fibril formation in a calcium-free condition *in vitro*^22^, Coker *et al.*^24^ suggested that not the amyloid recognition site on the B face of SAP, but the A face and/or the edge of the SAP pentamer has chaperone activity to enhance the refolding of client proteins. As SAP decamer with the B-face-to-B-face topology potently inhibited amyloid fibril formation from both Aβ(1-40) and D76N β2-m in a calcium-free buffer (Fig. 2 and Supplementary Fig. S1), the A face (and/or the edge) of the SAP pentamer may have the anti-amyloidogenic activity *in vitro*. We showed that in the presence of Ca^2+^, SAP first inhibited, then significantly accelerated D76N β2-m amyloid fibril formation (Fig. 5). Electron microscopy revealed the surfaces of D76N β2-m fibrils coated with doughnut-shaped pentameric SAP (Fig. 5). Moreover, when EDTA was spiked to the SAP-containing MES-Ca samples during the lag phase, EDTA switched off the pro-amyloidogenic effect of SAP but kept its anti-amyloidogenic effect intact (Fig. 6d–f). These data suggest that SAP (possibly A face) first inhibits the nucleation of D76N β2-m by interacting with soluble oligomers and partially unfolded monomers, while after mature amyloid fibrils are formed in the reaction mixture, high-affinity binding of the B face to the surface of amyloid fibrils^2^,^17^,^18^,^19^ prevails over the anti-amyloidogenic activity of the A face, leading to the accelerated amyloid fibril growth. To the best of our knowledge, this is the first study showing both the A-face-dependent anti-amyloidogenic activity and the B-face-dependent pro-amyloidogenic activity of SAP in a single experimental frame, proposing a model that the pro- and anti-amyloidogenic activities of SAP are not mutually exclusive, but reflect two sides of the same coin, i.e., the B and A faces, respectively.

Similar bidirectional activities for amyloidogenesis were observed for clusterin^27^,^37^,^47^. Yerbury *et al.*^37^ reported that clusterin exerts pro-amyloidogenic effects under conditions in which the substrate protein is present at a very large molar excess, while at much higher but still substoichiometric levels (e.g., a molar ratio of clusterin:substrate=1:10), it potently inhibits amyloid formation and provides substantial cytoprotection. Likewise, although clusterin was reported to enhance the clearance rate of Aβ42 across the blood-brain barrier^48^, DeMattos *et al.*^47^ reported that in a mouse model of AD, clusterin knockout reduced the fibrillar Aβ deposits and the neuritic dystrophy associated with the deposited amyloid. These data may indicate that ECs could exert multifaceted effects on the protein misfolding/aggregation and amyloid deposition, depending on the molecular environments where they exist. Future studies are eagerly awaited to elucidate the molecular mechanisms of these multifaceted activities of individual ECs.

To the best of our knowledge, this is the first study to indicate the anti-amyloidogenic, anti-aggregation activity of CRP (possibly the A face). Although CRP potently inhibited both Aβ(1-40) and D76N β2-m amyloid fibril formation in a Ca^2+^-independent manner (Figs 2 and 3, Supplementary Figs S1 and S2), the biological significance of these data should be considered carefully. Except for a few reports^13^,^14^,^15^, there are no convincing reports indicating the colocalization of CRP with amyloid deposits *in vivo*. This may be because the B face of CRP, unlike SAP, does not bind to the surface of amyloid fibrils Ca^2+^-dependently^19^. We propose a working hypothesis that the *in vitro* anti-amyloidogenic activity of CRP might reflect the general chaperone activity of CRP to maintain extracellular proteostasis *vide infra*.

Recently, various species of misfolded and aggregated proteins are considered to activate innate immune responses and inflammation, thus contributing to the pathophysiolosy of amyloidoses and other protein misfolding diseases^49^,^50^. The receptors of innate immune system recognize the hydrophobic portions of misfolded and aggregated proteins as universal damage-associated molecular patterns^51^. Considering that inflammation is a state in which host proteins are exposed to various stresses, including acidic pH^10^,^11^,^12^ and oxidants^42^, protein misfolding/aggregation and inflammation may generate a vicious circle^27^. Based on this scenario, Wilson’s group proposed that α2M and other ECs are specialized to prevent the extracellular accumulation of misfolded and potentially pathogenic proteins during the activation of innate immune system^27^,^42^. SAP inhibited the heat-induced amorphous aggregation of human GST (Fig. 8). Thus, the present study may indicate that CRP and SAP may maintain the proteostasis of the inflammatory site by inhibiting the aggregation of unfolded/misfolded proteins. This model is consistent with the hypothesis that pentameric CRP protects against toxic conditions caused by protein misfolding and aggregation in acidic inflammatory environments^8^,^9^, and is consistent with the observation that the A face and/or the edges of pentameric human SAP exhibits chaperone activity to enhance the refolding of denatured proteins^24^. Recently, Thiele *et al.*^7^ reported that pentameric CRP dissociates into monomeric CRP at sites of inflammation, which is then deposited and via its proinflammatory effects acts to amplify and localize inflammation. In the absence of Ca^2+^, CRP partially dissociated into a monomer at 72 h (Fig. 1c) and was a better anti-amyloidogenic protein with Aβ(1-40) than SAP decamer (Fig. 2b). Thus, the dissociation of CRP at sites of inflammation might enhance its chaperone activity as in the case of α2M, which also dissociates into dimers at inflammatory sites and exhibits enhanced chaperone activity^28^,^42^.

In conclusion, we obtained new insight into the chaperone activity of pentraxins, proposing that 1) classical pentraxins (CRP, SAP) may be a member of extracellular chaperones, and 2) the pro- and anti-amyloidogenic activities of SAP are not mutually exclusive, but reflect two sides of the same coin. As a therapeutic strategy, Pepys *et al.*^52^ developed a competitive inhibitor of SAP binding to amyloid fibrils, called CPHPC. CPHPC also crosslinked and dimerized SAP molecules with B-face-to-B-face stacking, leading to their very rapid clearance by the liver and marked depletion of circulating human SAP. This mechanism of drug action potently removed SAP from human amyloid deposits in the tissues. CPHPC treatment is expected to be effective because this treatment efficiently eliminates the pro-amyloidogenic activity of the B face of SAP (Figs 5 and 6). During the rapid removal of CPHPC-stabilized decameric SAP from the circulation and the tissues, the A face of SAP may capture misfolded amyloidogenic intermediates and eliminate these species from the circulation and the tissues, further contributing to the treatment of amyloidosis.

## Methods

### Materials

Human CRP and SAP were isolated by the team of Professor Mark B. Pepys (Wolfson Drug Discovery Unit, Centre for Amyloidosis and Acute Phase Proteins, Division of Medicine, University College London) as described elsewhere^53^,^54^,^55^. CRP and SAP were obtained with full informed consent from each individual donor. All individuals were paid donors in the USA, where the plasma was collected at centers approved by the UK Department of Health. Donor selection, donor examination and plasma collection were performed according to standards and/or requirements set by the UK Department of Health, in accordance with the European Pharmacopoeia monograph ‘Human Plasma for Fractionation’. No additional or specific ethical committee approvals were sought as these are not required for experimental *in vitro* use of plasma proteins isolated from donor plasma obtained with informed consent and processed under the prevailing strict regulatory guidelines specified here. AGP and GST were obtained from Sigma. MDH and LDH were obtained from Roche. Aβ(1-40) was purchased from Peptide Institute, Inc. (Osaka, Japan).

### Construction of an expression plasmid for D76N β2-m

The primer sequence 5′-CC ACT GAA AAA AAT GAG TAT GCC TGC-3′ was used for mutagenesis of aspartate at position 76 into asparagine. The primer and complementary primer were purchased from Sigma. We introduced site-directed mutations with these primers using the QuikChange site-directed mutagenesis kit (Stratagene) into a previously constructed plasmid (pAED4) containing a wild-type β2-m sequence^56^. To obtain a larger amount of plasmids, *Escherichia coli* XL-1 Blue cells were transformed with the plasmids and the transformants were selected using ampicillin on LB plates. A Wizard Plus Miniprep kit (Promega) was employed to extract the plasmids. We verified the sequence of the mutant in the plasmids using a DNA sequencer, the Applied Biosystems 3130 Genetic Analyzer (Thermo Fisher Scientific Inc., Waltham, MA). Recombinant human D76N β2-m was expressed using an *Escherichia coli* expression system and purified^56^.

### Gel filtration chromatography

Gel filtration analysis of CRP and SAP was carried out on a Superose 12 column using the ÄKTA pure chromatography system (GE Healthcare UK Ltd., UK). The buffers used were as follows: Tris-EDTA buffer, comprised of 50 mM Tris-HCl (pH 7.5), 150 mM NaCl, and 10 mM EDTA; Tris-Ca buffer, comprised of 50 mM Tris-HCl (pH 7.5), 150 mM NaCl, and 2 mM CaCl_2_; MES-Ca buffer, comprised of 50 mM MES-NaOH (pH 7.0), 500 mM NaCl, and 2 mM CaCl_2_; and MES-EDTA buffer, comprised of 50 mM MES-NaOH (pH 7.0), 500 mM NaCl, and 10 mM EDTA. CRP and SAP at 1.5 μM were incubated in Tris-EDTA, Tris-Ca, MES-Ca, or MES-EDTA buffer at 37 °C for 0 or 72 h. Then 300 μL aliquots were applied on a column equilibrated and eluted with the same buffer at 15 °C. The flow rate was 0.5 mL/min and elution was monitored by absorbance at 280 nm. The assembly states of CRP and SAP were determined using gel filtration standards (bovine thyroglobulin, 670 kDa, bovine γ-globulin, 158 kDa, chicken ovalbumin, 44 kDa, horse myoglobin, 17 kDa, and vitamin B_12_, 1.4 kDa; Catalog #151–1901, Bio-Rad, Hercules, CA) applied separately.

### Aβ(1-40) amyloid fibril formation and ThT assay

The reaction mixture (100 μL) that contained 25 μM Aβ(1-40), 0–1.25 μM CRP, SAP, or AGP, Tris-EDTA (pH 7.5), Tris-Ca (pH 7.5), or MES-Ca buffer (pH 7.0), and 5 μM ThT was incubated at 37 °C without shaking in a 96-well plate (675096, Greiner Bio-One GmbH, Frickenhausen, Germany) sealed with a sealing film (676070, Greiner Bio-One GmbH). After removing the sealing film each time, the top ThT fluorescence was measured using a SpectraMax M5 microplate reader (Molecular Devices, Sunnyvale, CA) at 25 °C with excitation at 445 nm and emission at 490 nm.

### D76N β2-m amyloid fibril formation and ThT assay

The reaction mixture (200 μL) that contained 30 μM D76N β2-m, 0–1.5 μM CRP, SAP, or AGP, Tris-EDTA (pH 7.5), Tris-Ca (pH 7.5), MES-Ca (pH 7.0), or MES-EDTA buffer (pH 7.0), and 5 μM ThT was incubated with shaking (800 rpm) at 37 °C in a 96-well plate (237105, Thermo Fisher Scientific, Nunc A/S, Roskilde, Denmark) sealed with sealing film. After removing the sealing film each time, the top ThT fluorescence was measured using a SpectraMax M5 microplate reader at 25 °C with excitation at 445 nm and emission at 485 nm. In some experiments, EDTA at the final concentration of 10 mM was spiked to the samples containing MES-Ca buffer (pH 7.0) at the end of or during the lag phase of ThT fluorescence (see Fig. 6).

### Transmission electron microscopy

The sample was spread on carbon-coated grids, negatively stained with 1% phosphotungstic acid (pH 7.0), and examined under a Hitachi H-7650 electron microscope with an acceleration voltage of 80 kV.

### Enzyme-linked immunosorbent assay (ELISA)

We used a 96-well ELISA plate kit (Sumitomo Bakelite). Each well was first coated with 100 μL of 20 μg/mL CRP or SAP dissolved in the coating buffer supplied by the manufacturer. After washing three times with Tris-EDTA (pH 7.5), Tris-Ca (pH 7.5), or MES-Ca buffer (pH 7.0) containing 0.05% Tween 20, 100 μl of 25 μM Aβ(1-40) or 30 μM D76N β2-m in each buffer containing 0.05% Tween 20 was added to the wells and incubated for 1 h at 25 °C. Yerbury *et al.*^37^,^38^ reported that prefibrillar species on the fibril-forming pathway are enriched in the reaction mixture at the beginning of the growth phase (i.e., at the end of the lag phase). Thus, the reaction mixture that contained 25 μM Aβ(1-40) or 30 μM D76N β2-m, Tris-EDTA (pH 7.5), Tris-Ca (pH 7.5), or MES-Ca buffer (pH 7.0), and 5 μM ThT was incubated at 37 °C as described above and ThT fluorescence monitored. The fresh reaction mixture and the mixture at the beginning of the growth phase (aggregated mixture) (see Figs 2, 3, 4, 5 and Supplementary Figs S1 and S2) were spiked with Tween 20 (final 0.05%), added to the wells and incubated for 1 h at 25 °C. After washing three times with each buffer that contained 0.05% Tween 20, bound Aβ(1-40) and D76N β2-m were detected with anti-human Aβ(1-40) (Sigma) and β2-m (Dako) antibodies (1:1,000), respectively, and horseradish peroxidase-conjugated anti-rabbit immunoglobulins antibody (1:2,000) (Dako) followed by color development using 3,3′,5,5′-tetramethylbenzidine as the peroxidase substrate (Bio-Rad). The absorbance was measured at 450 nm in a SpectraMax M5 microplate reader (Molecular Devices, Sunnyvale, CA). The absorbance of the well which was not coated with pentraxins was subtracted from each data.

### Crosslinking experiments with BS^3^ and western blotting analysis

Crosslinking reaction was performed in the reaction mixture of Aβ(1-40) and D76N β2-m amyloid fibril formation in the presence of Ca^2+^. First, the reaction mixture containing 25 μM Aβ(1-40) or 30 μM D76N β2-m, PIPES-Ca buffer (50 mM PIPES-NaOH (pH 7.5), 150 mM NaCl, and 2 mM CaCl_2_) or MES-Ca buffer (pH 7.0), and 5 μM ThT was incubated at 37 °C as described above and ThT fluorescence monitored. At 0 h and at the beginning of the growth phase, the reaction mixture in PIPES-Ca buffer was spiked with 1:20 CRP and that in MES-Ca buffer was spiked with 1:20 SAP, and both mixtures were incubated for 30 min at 37 °C. BS^3^ (Thermo Fisher Scientific), an amine-reactive crosslinking reagent, was then added to the mixture at a final concentration of 5 mM, incubated for additional 45 min at 37 °C, and the crosslinking reaction quenched with 50 mM Tris buffer (pH 7.5). Crosslinked products were separated by 3–10% gradient SDS-PAGE and the protein bands detected by Coomassie Brilliant Blue staining. After separate SDS-PAGE performed in parallel, the proteins were transferred to PVDF membranes (Bio-Rad) and the membranes blocked with 1% casein. After washing three times with a washing buffer (20 mM Tris-HCl (pH 7.6), 137 mM NaCl, and 0.1% Tween 20), bound Aβ(1-40) and D76N β2-m were detected with anti-human Aβ(1-40) (Sigma) and β2-m (Dako) antibodies (1:1,000), respectively, and horseradish peroxidase-conjugated anti-rabbit immunoglobulins antibody (1:2,000) (Dako) followed by enhanced chemiluminescence with BM Chemiluminescent Blotting substrate (Roche).

### Aggregation of client proteins and turbidity assay

The reaction mixture (100 μL) containing 8.5 μM human GST (47 kDa), 2 μM porcine MDH (70 kDa), or 4 μM rabbit LDH (140 kDa), 0–8 μM SAP, and MES-Ca (pH 7.0) or Tris-EDTA buffer (pH 7.5) was incubated at 43 °C without shaking in a 96-well plate (675096, Greiner Bio-One GmbH, Frickenhausen, Germany) sealed with sealing film (676070, Greiner Bio-One GmbH). After removing the sealing film each time, the turbidity was measured using a SpectraMax M5 microplate reader (Molecular Devices, Sunnyvale, CA) at 25 °C with absorbance at 360 nm. The absorbance of the well at 0 h was subtracted from each data.

### Statistical analysis

Statistical analysis was performed by the unpaired Student’s t-test. Values with p < 0.05 were considered statistically significant.

## Additional Information

**How to cite this article**: Ozawa, D. *et al.* Multifaceted anti-amyloidogenic and pro-amyloidogenic effects of C-reactive protein and serum amyloid P component *in vitro.* *Sci. Rep.* **6**, 29077; doi: 10.1038/srep29077 (2016).

## Supplementary Material

Supplementary Information
