# Supplementary material for: Multifaceted anti-amyloidogenic and pro-amyloidogenic effects of C-reactive protein and serum amyloid P component in vitro
Source: Sci Rep. 2016 Jul 6;6:29077. doi: 10.1038/srep29077 (PMC4933921; doi:10.1038/srep29077)
Supplement: Supplementary Information [file srep29077-s1.pdf]

Supplemental Data

"Multifaceted anti-amyloidogenic and pro-amyloidogenic effects of C-reactive protein and serum amyloid P component in vitro"

Daisaku Ozawa, Ryo Nomura, P. Patrizia Mangione, Kazuhiro Hasegawa, Tadakazu Okoshi, Riccardo Porcari, Vittorio Bellotti & Hironobu Naiki

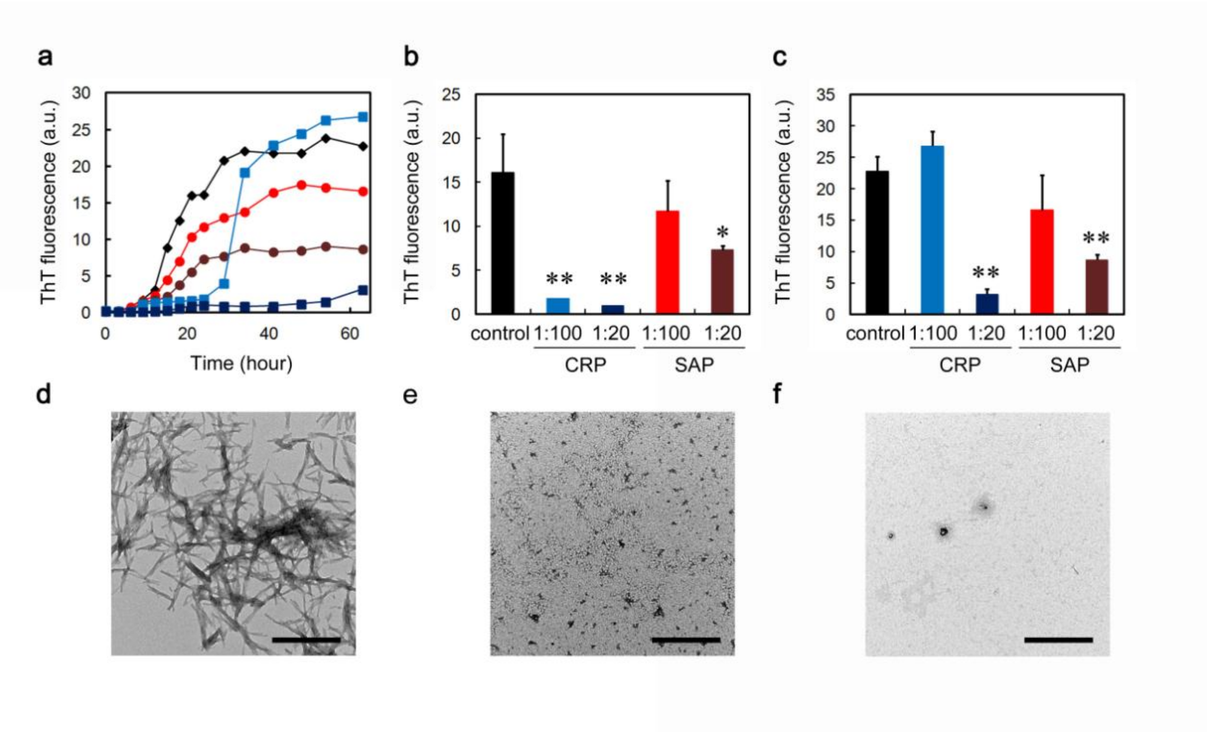

Supplementary Figure S1. Effects of CRP and SAP on D76N β2-m amyloid fibril formation in Ca^{2+}-free Tris-EDTA buffer. (a) Time course of fibril formation monitored by ThT fluorescence in the absence (black diamond) or presence of 1:100 (molar ratio of CRP to D76N β2-m) (blue square), or 1:20 (dark blue square) CRP, or 1:100 (red circle), or 1:20 (dark brown circle) SAP. Each point represents the average of three independent incubations. Representative data of three independent experiments are shown. (b, c) ThT fluorescence of each sample at 24 (b) or 63 h (c) in (a). The data are mean ± SD of three independent incubations. Statistical analysis was performed by unpaired Student's t-test. *$P < 0.05$, **$P < 0.01$ vs. control. (d-f) Electron microscopy images of the samples of fibril formation. The sample prepared in the absence (d) or presence of 1:20 CRP (e) or SAP (f) was incubated at 37 °C for 18 h. The scale bar represents 0.5 μm.

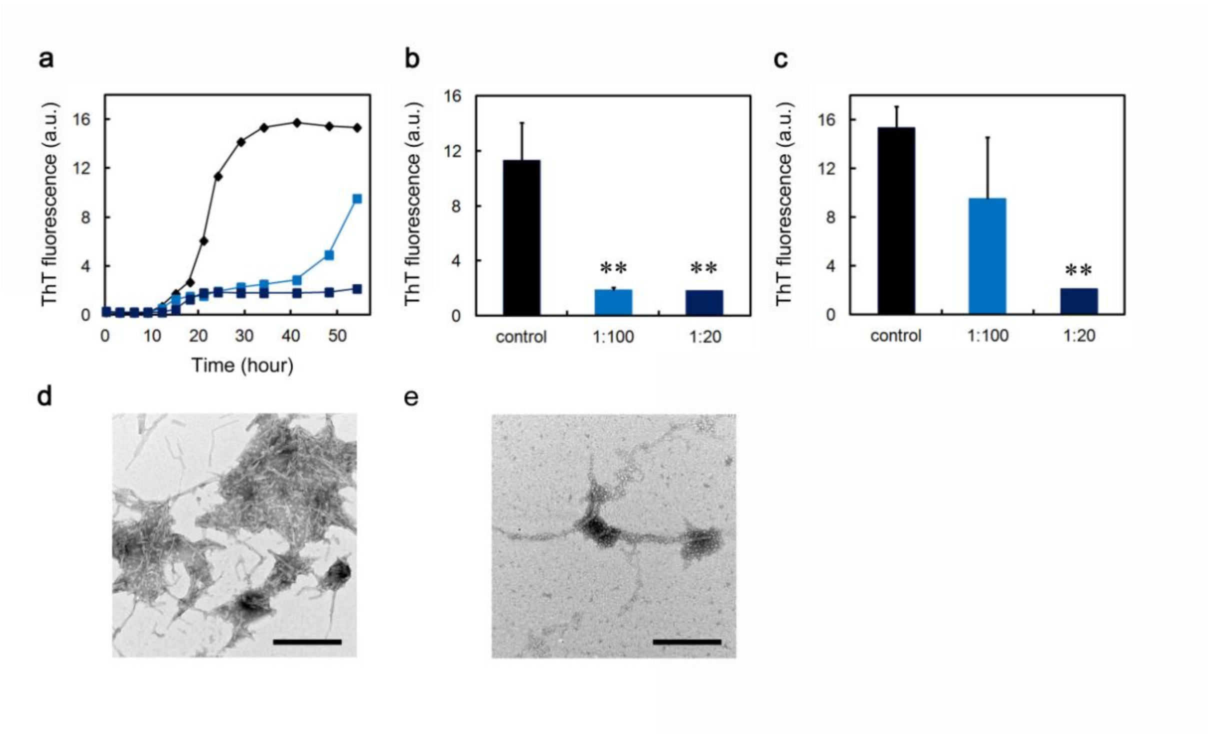

Supplementary Figure S2. Effect of CRP on D76N β2-m amyloid fibril formation in Tris-Ca buffer. (a) Time course of fibril formation monitored by ThT fluorescence in the absence (black diamond) or presence of 1:100 (molar ratio of CRP to D76N β2-m) (blue square), or 1:20 (dark blue square) CRP. Each point represents the average of three independent incubations. Representative data of three independent experiments are shown. (b, c) ThT fluorescence of each sample at 24 (b) or 54 h (c) in (a). The data are mean ± SD of three independent incubations. Statistical analysis was performed by unpaired Student's t-test. **P < 0.01 vs. control. (d, e) Electron microscopy images of the samples of fibril formation. The sample prepared in the absence (d) or presence of 1:20 CRP (e) was incubated at 37 °C for 34 h. The scale bar represents 0.5 μm.

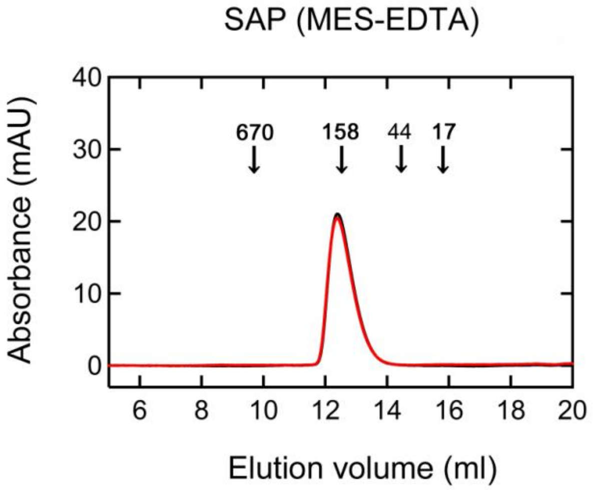

Supplementary Figure S3. Analysis of molecular weight distribution of SAP in MES-EDTA buffer by gel filtration chromatography. SAP at 1.5 μM was incubated in MES-EDTA buffer (pH 7.0) at 37 °C for 0 (black line) or 72 h (red line), then 300 μL aliquots were applied on a column equilibrated and eluted with the same buffer at 15 °C. Elution was monitored by absorbance at 280 nm. Arrows in each figure indicate the elution volumes of molecular weight markers (kDa).

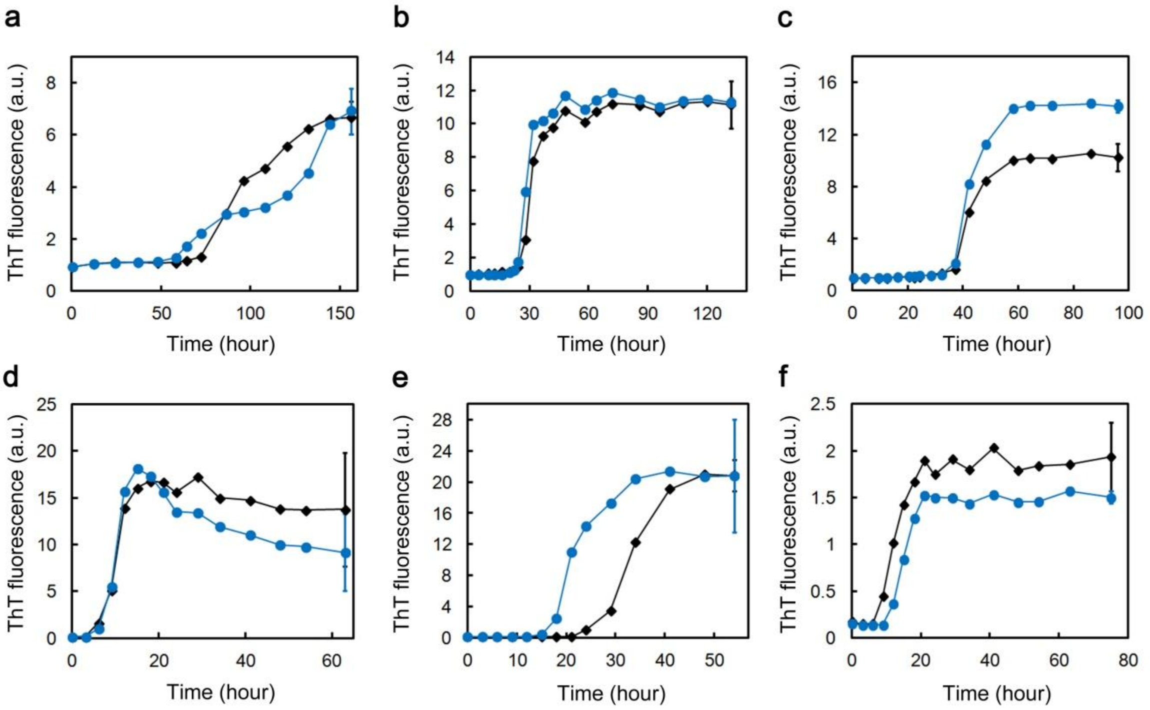

Supplementary Figure S4. Effect of AGP on Aβ(1-40) and D76N β2-m amyloid fibril formation in Tris-EDTA, Tris-Ca, and MES-Ca buffers. (a-f) Time courses of Aβ(1-40) (a-c) and D76N β2-m (d-f) fibril formation monitored by ThT fluorescence in the absence (black diamond) or presence of 1:20 AGP (blue circle) in Tris-EDTA (a, d), Tris-Ca (b, e), or MES-Ca buffer (c, f). Each point represents the average of three independent incubations. At each endpoint, the data are mean ± SD of three independent incubations. Representative data of three independent experiments are shown.

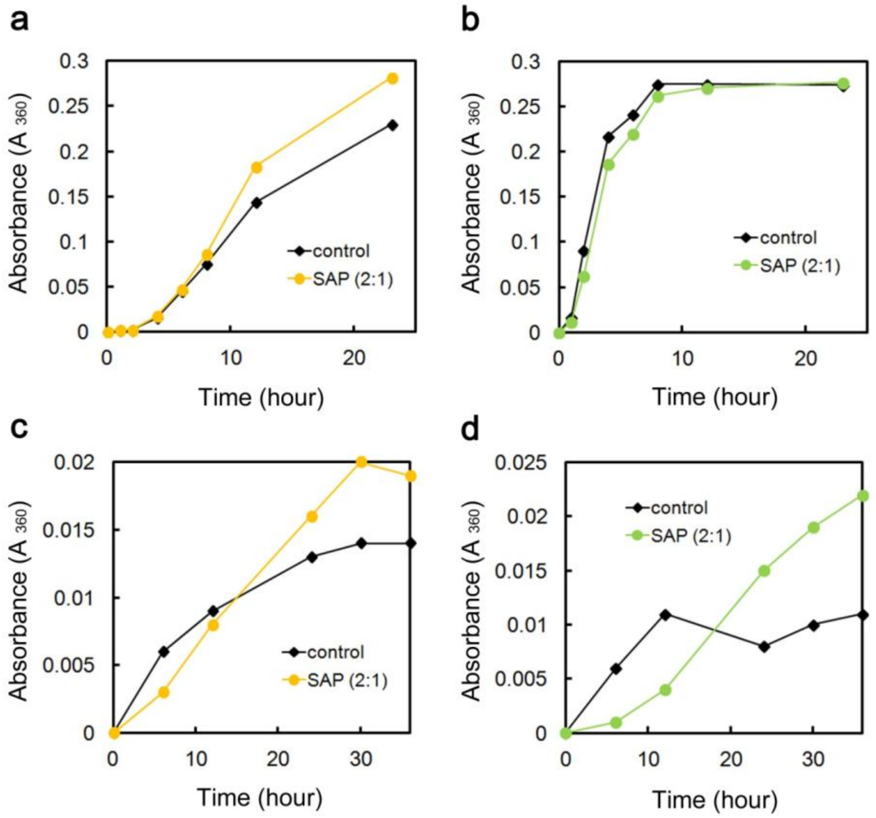

Supplementary Figure S5. Effect of SAP on heat-induced aggregation of MDH and LDH in MES-Ca and Tris-EDTA buffers. Time course of MDH (a, b) and LDH (c, d) aggregation monitored by turbidity in the absence (black diamond) or presence of 2:1 (molar ratio of SAP to MDH/LDH) SAP in MES-Ca buffer (orange circle) (a, c), or 2:1 SAP in Tris-EDTA buffer (right green circle) (b, d) at 43 °C. Each point represents the average of three independent incubations. Representative data of two independent experiments are shown.
